# Why are you looking at me like that? Intolerance of uncertainty and emotional face processing in social anxiety disorder

**DOI:** 10.1016/j.nicl.2026.104069

**Published:** 2026-09-25

**Authors:** Antonia Küttner, Esther Zwiky, Philine König, Konrad Schöniger, Robin Kamrla, Gina Sophie Scheiwe, Julia Andreev, Johanna Elea Hamann, Annika Brückner, Sina Pletsch, Janine Selle, Alexander Gussew, Andreas Deistung, Walter Alexander Wohlgemuth, Ronny Redlich

**Affiliations:** a Department of Psychology, University of Halle, Germany; b German Center for Mental Health (DZPG), Halle-Jena-Magdeburg, Germany; c Center for Intervention and Research on Adaptive and Maladaptive Brain Circuits Underlying Mental Health (CIRC), Halle-Jena-Magdeburg, Germany; d Institute for Translational Psychiatry, University of Münster, Germany; e University Clinic and Policlinic for Radiology, University Hospital Halle, University Medicine Halle, Germany; f Halle MR Imaging Core Facility, Medical faculty of University of Halle, Germany

**Keywords:** Social anxiety disorder, Intolerance of uncertainty, fMRI, Functional connectivity, Face processing, Insula

## Abstract

**Background:**

Social Anxiety Disorder (SAD) is a prevalent mental disorder characterized by altered processing of social stimuli. Intolerance of Uncertainty (IU) has been linked to social anxiety and influences the perception of social information. Yet, the neural correlates of IU in SAD during the processing of emotional stimuli remain insufficiently understood.

**Methods:**

In this study, fMRI data were collected from 25 individuals with SAD and 25 healthy controls (HC), who completed a face processing task. Differential associations between the IU subdimensions *prospective IU*, *inhibitory IU*, and *burden due to IU,* brain activity, and functional connectivity were analyzed using full-factorial models. Functional connectivity measures focused on the insula.

**Results:**

For *prospective IU*, analysis of functional activity revealed an interaction effect in parietal regions and the insula (*p*_*TFCE-*FWE_ ≤ 0.031), driven by a positive association between IU and activation in the HC group. Concurrently, *prospective IU* was negatively associated with insular functional connectivity across both groups (*p*_TFCE-FWE_ ≤ 0.031), suggesting reduced insular integration independent of diagnosis. A distinct pattern emerged for *burden due to IU*, showing a negative association with precuneus and posterior cingulate gyrus functional activity across both groups (*p*_TFCE-FWE_ = 0.014). No significant effects were found for *inhibitory IU*.

**Conclusions:**

These findings indicate both disorder-specific and shared neural associations of IU. In particular, *prospective IU* may hold clinical relevance in SAD, as it is linked to reduced insular connectivity across groups but to increased neural activity exclusively in HC, possibly pointing toward neural vulnerabilities that could inform future approaches to the diagnosis and treatment of SAD.

## Highlights

• *Prospective IU* is linked to reduced insular connectivity across groups, but to increased activity in healthy controls.
• *Burden due to IU* was negatively associated with activity in the precuneus and posterior cingulate gyrus across both groups.

## 1 Introduction

Navigating social environments is a complex yet evolutionarily fundamental function of the human brain. Accordingly, specialized neural circuits, referred to as the social brain, have evolved to support social cognition and behavior (Frith, 2007). These networks enable the rapid detection of social stimuli and the extraction of predictive information about others' intentions and actions. Although such neural sensitivity is fundamentally adaptive, it may also confer vulnerability by amplifying the perceived ambiguity or threat associated with social stimuli.

This vulnerability may be particularly pronounced in individuals with social anxiety disorder (SAD), a prevalent mental disorder characterized by excessive fear of situations involving potential judgment or evaluation by others, accompanied by the avoidance of or intense distress in these situations (Falkai and Wittchen, 2015). Altered social-emotional processing, reflected in an attentional bias toward stimuli perceived as threatening and those specific to the disorder (Günther et al., 2021; Pei et al., 2023), is a core feature of SAD.

Neurofunctional models of SAD describe a complex interplay between heightened activity in core fear-related regions, including the amygdala, insula, anterior cingulate cortex (ACC), and prefrontal cortex, together with increased activation in medial parietal and occipital regions and reduced connectivity between parietal, limbic, and executive network regions (Brühl et al., 2014). Recent resting-state findings also show widespread abnormalities in large-scale functional network connectivity, supporting an imbalance between exaggerated bottom-up threat processing and compromised top-down regulation (Zhang et al., 2023).

Beyond general social situations, functional neuroimaging research on SAD has extensively focused on emotional facial expressions as disorder-relevant and salient stimuli. Face-to-face interactions represent a central context for anxiety and avoidance in SAD, with patients often fearing the perception of negative emotional expressions in their interaction partners (Gentili et al., 2016). Hyperactivation during face perception in brain regions involved in emotion processing, again emphasizing the role of the amygdala, insula, and ACC, as well as in visual processing areas (for Meta-Analysis, see Gentili et al., 2016), is consistently reported. Extending these findings, social anxiety is positively associated with insula and prefrontal cortex activity during the perception of angry faces, suggesting a dimensional influence of social anxiety on neural processing (Carré et al., 2014). Beyond regional activation differences, evidence of altered functional connectivity during face perception (Pantazatos et al., 2014) further underscores the importance of extending the neural perspective on SAD to the network level. Taken together, these findings suggest heightened sensitivity to, and a potential attentional bias toward, emotional facial expressions, pointing to disorder-related alterations in emotional face processing. These alterations may impair the adaptive use of socially relevant information conveyed by facial expressions and, in turn, contribute to difficulties in social interaction.

A central characteristic of social stimuli, such as facial expressions, may be their inherent uncertainty and ambiguity, as their interpretation and implications depend on a multitude of factors, including contextual information, prior experiences, and the dynamics of the relationship between interaction partners (Aviezer et al., 2017; FeldmanHall and Shenhav, 2019). A reduced capacity to tolerate such uncertainty and a tendency to respond negatively to situations perceived as ambiguous or lacking sufficient information, referred to as intolerance of uncertainty (IU), has been proposed as a key transdiagnostic factor in the development and maintenance of anxiety disorders, including SAD (Carleton, 2012; Carleton et al., 2010). In SAD, elevated levels of IU as well as associations between social anxiety symptoms and IU have been demonstrated consistently (Boelen and Reijntjes, 2009; Carleton, 2012; Carleton et al., 2010). From an IU perspective, core features of SAD, such as the use of safety behaviors and heightened self-focused attention, may serve to reduce or manage the unbearable feelings of uncertainty experienced in social situations (Carleton et al., 2010). Given that IU is presumed to manifest across cognitive, behavioral, and emotional domains (Freeston et al., 1994; Gerlach et al., 2008), a multidimensional conceptualization may help clarify how its distinct facets contribute to disorder-related mechanisms. In line with previous work defining IU as a multifaceted construct with distinct neural correlates (Küttner et al., 2025; Nelson et al., 2016), the present analyses differentiated between *prospective IU,* characterized by a desire for predictability and cognitive effort to reduce uncertainty, *inhibitory IU*, reflecting behavioral inhibition in uncertain situations (Carleton et al., 2007), and *burden due to IU*, encompassing feelings of discomfort, anxiety, and stress when confronted with uncertainty (Gerlach et al., 2008).

Beyond its role in social anxiety symptomatology, IU itself appears to modulate the processing of and behavioral responses to social stimuli. Individuals high in IU exhibit an attentional bias toward angry faces in uncertain environments, as evidenced by eye-tracking (Morriss et al., 2018), impaired extinction learning in response to social threat (Wake et al., 2021), and reduced amygdala habituation to emotional faces (Jeon et al., 2026). Taken together, these findings point to a broader link between IU, uncertainty processing, and fear-related brain regions, supported by neuroimaging findings of altered activity in the amygdala and insula relative to IU (Tanovic et al., 2018). The insula, in particular, may serve as a key region in the context of unpredictability, showing heightened activation to uncertain stimuli regardless of valence, with insular activity (Gorka et al., 2016; Shankman et al., 2014) and functional connectivity (Deserisy et al., 2020; Radoman and Gorka, 2023) modulated by IU.

As a central node of the salience network, the insula integrates self-referential and sensory information, facilitates attentional shifts toward salient cues, and coordinates corresponding affective responses (Seeley, 2019; Seeley et al., 2007). In SAD, heightened insular activity during face processing (Freitas-Ferrari et al., 2010; Klumpp et al., 2011) and exposure to disorder-related scenes (Heitmann et al., 2016) therefore likely reflects amplified interoceptive signaling, a mechanism that may underlie the heightened focus on the self and dysfunctional allocation of attention characteristic of the disorder.

In conclusion, IU, SAD, and face perception are conceptually and empirically intertwined, sharing overlapping neurobiological foundations and processes. However, to our knowledge, no studies have directly investigated how different subdimensions of IU relate to the neural processing of social-emotional stimuli, such as emotional faces, and whether these relationships differ between individuals with and without SAD. Building on the findings discussed above, it can be inferred that both IU and the presence of social anxiety symptoms may influence the extent to which faces depicting negative emotions are perceived as threatening or uncertainty-eliciting. Accordingly, it is expected that individuals with and without SAD will differ in their neural responses to these stimuli in relation to their levels of IU.

Elucidating how social stimuli are processed, integrated, and evaluated in SAD may provide insight into factors that increase vulnerability to, or protect against, the manifestation of the disorder. Further, understanding the neural interaction between IU, SAD, and face perception may open new avenues for treatment, particularly as both IU (Katz et al., 2017) and brain activity during emotional face processing (Doehrmann et al., 2012) have previously been identified as predictors of symptom reduction over the course of psychotherapy for social anxiety.

Given the evidence of elevated levels of IU in patients with SAD (Boelen and Reijntjes, 2009; Carleton, 2012; Carleton et al., 2010) and alterations in the processing of emotional stimuli in this patient group (Carré et al., 2014; Gentili et al., 2016; Pantazatos et al., 2014), the present study aimed to examine how IU subdimensions relate to brain activity and functional connectivity during emotional face perception.

In light of the insula's involvement in uncertainty-related as well as social-emotional processes, particular emphasis was placed on its functional connectivity. Specifically, this study addressed three aims: (1) to examine whether the IU subscales *prospective IU, inhibitory IU*, and *burden due to IU* are associated with distinct patterns of neural activation during emotional face processing; (2) to examine whether these IU subscales are associated with distinct patterns of insular functional connectivity; and (3) to explore whether these activation and connectivity patterns differ between individuals with SAD and healthy controls (HC). Given the scarcity of research on the neural correlates of IU in SAD, we adopted an exploratory approach to these questions, without specifying directional hypotheses. By integrating dimensional measures of IU subfactors with brain functional activity and seed-based connectivity analyses, this study seeks to clarify the neurobiological mechanisms linking uncertainty aversion, social-emotional perception, and social anxiety symptomatology.

## 2 Materials and methods

### 2.1 Participants and study design

The sample included *n* = 25 adult individuals with SAD and *n* = 25 HC, matched for age and gender. Individuals in the SAD group were recruited from the University of Halle-Wittenberg outpatient clinic and the Institute for Psychological Psychotherapy in Halle. HC were recruited through public notices and newspaper advertisements. Exclusion criteria for both groups included neurological abnormalities, prior traumatic head injuries, organic mental disorders, dementia, chronic medical conditions, substance or alcohol abuse and dependence, or MRI contraindications. Additionally, HC were required to have no lifetime psychiatric disorder, as assessed with the German Structured Clinical Interview for DSM-5 (SCID-5-CV (Beesdo-Baum et al., 2019)) and to be free of psychotropic medication. All individuals with SAD met DSM-5 criteria for the disorder, confirmed by the SCID-5-CV. Participation took place prior to the initiation of cognitive behavioral therapy: after the first consultation or during its initial trial sessions. 23 patients presented with comorbid disorders, and six patients were concurrently receiving psychiatric medication. To quantify medication effects, a medication load index was calculated following the approach of Hassel et al. (2008). Detailed information on comorbidities and medication load is provided in Table 1.

**Table 1:** Sociodemographic and clinical characteristics (*N* = 50).

	SAD Sample *(n = 25)*		HC Sample *(n = 25)*		*p-*value	Effect size
	*Mean*	*SD*	*Mean*	*SD*		
age in years^1^	25.84	4.42	24.92	5.36	0.117	0.04
Gender (m/f)	7/18	–	7/18	–	–	–
IU-18 total score^2^	59.16	16.31	38.20	10.32	<0.001	1.53
*Prospective IU*^2^	18.72	6.41	14.36	4.75	0.009	0.77
*Inhibitory IU*^2^	20.48	5.74	10.36	3.17	<0.001	2.18
*Burden due to IU*^2^	19.96	5.37	13.48	4.00	<0.001	1.37
STAI-T total score^1^	57.80	10.51	33.00	6.80	<0.001	0.63
BDI-II total score^1^	25.84	10.41	3.68	4.52	<0.001	0.60
SPS total score^1^	29.36	15.17	7.48	5.39	<0.001	0.55

Medication load index						
0 = absent	19 (76%)		–	–	–	–
1 = low	6 (24%)		–	–	–	–
2 = high	0 (0%)		–	–	–	–

Comorbidities, no. of patients^3^						
MDD	20 (80%)	–	–	–	–	–
PDD	5 (20%)	–	–	–	–	–
GAD	3 (12%)	–	–	–	–	–
ADHD	3 (12%)	–	–	–	–	–
Panic Disorder	2 (8%)	–	–	–	–	–
PTSD	2 (8%)	–	–	–	–	–
Other	4 (16%)	–	–	–	–	–

*Note.* SAD, Social Anxiety Disorder; HC, Healthy Controls; *SD*, Standard Deviation; IU-18, Uncertainty Intolerance Scale-18 (Gerlach et al., 2008); IU, Intolerance of Uncertainty; STAI-T, Spielberger State-Trait Anxiety Inventory (Laux et al., 1981); BDI-II, Beck Depression Inventory II (Beck et al., 1996); SPS, Social Phobia Scale (Ulrich et al., 1999), MDD; Major Depressive Disorder; PDD, Persistent Depressive Disorder; GAD, Generalized Anxiety Disorder; ADHD, Attention deficit hyperactivity disorder; PTSD, Posttraumatic Stress Disorder; ^1^*p*-values were obtained using Mann-Whitney *U* tests, effect sizes were calculated as η^2^; ^2^*p*-values were obtained using two-tailed *t*-tests, effect sizes were calculated as Cohen's *d;*^3^Comorbidities with a frequency > 1 are listed individually, all others are summarized as “other”, multiple entries per person are possible.

Data were collected from November 2022 to August 2025. All participants completed an fMRI session, SCID-5-CV and a set of self-report inventories, assessing intolerance of uncertainty (IU-18 (Gerlach et al., 2008)), social phobia (SPS (Ulrich et al., 1999)), depressive symptoms (BDI-II (Beck et al., 1996)) and trait anxiety (STAI-T (Laux et al., 1981)).

The IU-18 (Gerlach et al., 2008) is the German version of the Intolerance of Uncertainty Scale originally developed by Freeston et al. (1994). In addition to the commonly reported subscales, *prospective* and *inhibitory IU*, it includes the factor of *burden due to IU*, identified as a third core component of IU by Dugas et al. (1998). For the analyses, total scores for each subscale (six items per subscale) were used. Reported reliability was high in prior research (Cronbach's α = 0.80–0.85) (Gerlach et al., 2008), and was comparable in the current sample (Cronbach's α = 0.78–0.89). The Social Phobia Scale (SPS (Ulrich et al., 1999)) was used to assess social anxiety symptom severity with a focus on anxiety in situations where one's actions are or could be observed by others, such as public speaking, eating, or writing. Total scores and intercorrelations are reported in Table 1, Table 2.

**Table 2:** Spearman correlation coefficients between IU-18 subscales, social phobia, state anxiety and depression scores, separated for patients with SAD (*n* = 25) and HC (*n* = 25).

	Prospective IU		Inhibitory IU		Burden due to IU		STAI-T		BDI-II	
	SAD	HC	SAD	HC	SAD	HC	SAD	HC	SAD	HC
Inhibitory IU	0.706**	0.517*	–	–						
Burden due to IU	0.890**	0.640**	0.738**	0.761**	–	–				
STAI-T	0.481*	−0.048	0.445*	0.344	0.533*	0.327	–	–		
BDI-II	0.325	0.036	0.484*	0.443	0.357	0.346	0.841**	0.507**	–	–
SPS	0.621**	0.241	0.638**	0.381	0.547*	0.269	0.337	0.559**	0.328	0.188

*Note.* SAD, Social Anxiety Disorder; HC, Healthy Controls; IU-18, Uncertainty Intolerance Scale-18 (Gerlach et al., 2008); IU, Intolerance of Uncertainty; STAI-T, Spielberger State-Trait Anxiety Inventory (Laux et al., 1981); BDI-II, Beck Depression Inventory II (Beck et al., 1996); SPS, Social Phobia Scale (Ulrich et al., 1999); *p < .05; ** *p* < .001.

The studies involving human participants were approved by the Ethics Committee of the Faculty of Medicine at the University of Münster (amendment 2020–205-f-s), and the Ethics Committee of the Medical Faculty of Martin Luther University Halle-Wittenberg (approval number 2022–023). The studies were conducted in accordance with the Declaration of Helsinki, the local legislation, and institutional requirements. The participants provided their written informed consent to participate in this study and received financial compensation.

### 2.2 fMRI paradigm, data acquisition, and preprocessing

To examine neural reactivity during negative emotional face processing, a well-established fMRI paradigm was used (Hariri et al., 2002; König et al., 2026; Redlich et al., 2015). The task consisted of four blocks of a face-processing task alternating with five blocks of a sensorimotor control task. Each of these nine blocks contained six trials. During the face-processing task, participants viewed three faces from the Ekman stimulus set (Ekman, 1976), expressing either anger or fear, balanced for gender and emotion. During the sensorimotor control task, participants viewed three geometric shapes (circles and ellipses). To ensure stimulus processing, participants were instructed to match one of the two faces or shapes at the bottom of the screen with the target face or shape at the top via keystroke. Each block was preceded by a fixation cross for 1.5 s and an instruction to match faces or shapes for 2 s. In each trial, the trio was presented for 4 s with an inter-stimulus interval ranging from 1.5 to 5.5 s (*M* = 3.9 s) in the face-processing task, and a fixed inter-stimulus interval of 1.5 s in the sensorimotor control task. This resulted in a total block length of 47 s (face-processing task) and 35 s (sensorimotor control task), and an overall paradigm length of 363 s.

MR data were collected on a 3 T scanner (Magnetom Vida 3 T, Siemens, Erlangen, Germany) equipped with a 64-channel head/neck coil. Images were shown at the rear end of the scanner using a dedicated MR-compatible 40” LCD monitor (NordicNeuroLab, Norwegia). Seventy-six contiguous T2*-weighted images were acquired with 2D echo-planar-readout (EPI), repetition time (TR) = 1.6 s, echo time (TE) = 32 ms, flip angle (FA) = 90, and voxel size 2 × 2 × 2 mm. Partial parallel imaging (GRAPPA, *R* = 2, 54 reference lines) and simultaneous multi-slice (SMS) with a factor of 4 were applied to accelerate the acquisition. In addition, whole-head T1-weighted (T1w) MRI data sets were collected with a magnetization-prepared rapid gradient-echo (MP-RAGE) sequence (isotropic voxel size of 0.8 mm, TE = 2.24 ms, T*R* = 2400 ms, inversion time [TI] = 1030 ms, FA = 8°, acquisition matrix = 320 × 300 × 208, GRAPPA with R = 2 and 32 reference lines, TA = 6:38 min:s) for spatial normalization. Functional MRI data were realigned, unwarped, corrected for slice-timing differences, spatially normalized to the standard Montreal Neurological Institute space, and smoothed (Gaussian kernel, 6 mm full-width at half maximum) using Statistical Parametric Mapping (SPM12, Wellcome Department of Cognitive Neurology, London, UK; http://www.fil.ion.ucl.ac.uk/spm). Onsets and durations of the two experimental conditions (faces and shapes) were modeled with a canonical hemodynamic response function in the context of the general linear model. For each participant, one contrast image was generated in each individual fixed-effects first-level analysis comparing activation in response to fear-relevant faces with the shapes baseline (faces > shapes). The resulting contrast images were then entered into second-level random-effects group analyses.

### 2.3 Preprocessing for functional connectivity analyses

To investigate alterations in insular functional connectivity during emotional face processing, T2*-weighted MRI data were additionally preprocessed using the CONN toolbox (Whitfield-Gabrieli and Nieto-Castanon, 2012), including realignment with correction of susceptibility distortion interactions, slice timing correction, outlier detection, direct segmentation, MNI-space normalization, and smoothing. Potential outlier scans were identified using ART (Whitfield-Gabrieli et al., 2011) and data were denoised using a standard denoising pipeline including the regression of potential confounding effects. Only activation during the faces condition was further analyzed. Detailed information is provided in the Supplementary Material.

### 2.4 Statistical analyses

Statistical analyses of clinical data were performed using SPSS Statistics (version 28.0.1.1; IBM Corporation), and second-level analyses of fMRI data were conducted using SPM 12. Group differences in sociometric and questionnaire measures were examined using two-tailed *t*-tests, with Mann–Whitney *U* tests applied when the assumption of normality was violated (see Table 1). To examine the incremental validity of IU beyond depressive symptoms and trait anxiety in explaining social anxiety, hierarchical linear regression analyses predicting SPS total scores were conducted separately for patients with SAD and HC. In the first step, age and gender were entered as predictors; BDI-II and STAI-T total scores were additionally entered in the second step, followed by the IU-18 total score in the third step.

Regarding analyses of brain activity, group differences during face processing were assessed between SAD patients and HC at the whole-brain and region-of-interest (ROI) levels, as well as in the functional connectivity of the left and right insula, using two-sided *t*-tests (contrasts: HC > SAD, HC < SAD).

To assess dimensional effects of IU on brain activity, a separate full-factorial model was specified in SPM for each IU subscale (*prospective IU, inhibitory IU, burden due to IU*). Group (SAD patients vs. HC) was entered as a between-subjects factor, and the interaction term between group and the respective IU subscale was modeled. Analyses were conducted at the whole-brain level and within the bilateral whole insula as a region of interest.

Subsequently, to examine associations between bilateral insular functional connectivity and IU subscales, full-factorial models were specified analogously to those used in the functional activity analyses, yielding six separate models. For the functional connectivity analyses, the left and right insula were defined as separate seed regions, and brain-wide coactivation during the face-condition was modeled.

In all full-factorial models, an ANCOVA was conducted, and undirected interaction effects between group and IU subscales on brain activity or connectivity were examined. When a significant interaction was observed, follow-up subgroup regression analyses were conducted to clarify the direction of the associations. In the absence of a significant interaction, the main effects of IU subscales on brain activity or connectivity across the whole sample were evaluated.

To assess whether the findings differed when using the IU-18 total score rather than differentiating between IU subscales, the aforementioned analyses were repeated using the IU-18 total score. Employing the same analytical settings, the analyses were additionally repeated with the SPS to further examine associations between social anxiety symptoms and functional brain activity and connectivity.

In all SPM analyses, age and gender were modeled as covariates of no interest. In analyses addressing dimensional effects of IU, BDI-II, and STAI-T scores were modeled as covariates of no interest to account for potential confounding effects of comorbid symptomatology. To examine the unique effects of each IU subscale, the respective remaining IU subscales were additionally entered as covariates of no interest. Threshold-free cluster enhancement (TFCE) was applied, as implemented in the TFCE toolbox (http://dbm.neuro.uni-jena.de/tfce, version 232). Parameters were set to *e* = 0.4 and *h* = 2. A conservative family-wise error (FWE)-corrected threshold of *p* < .05 was established, obtained by 5000 permutations per test. The AAL-Atlas implemented in the Wake Forest University Pickatlas (Tzourio-Mazoyer et al., 2002) was used for cluster labeling and ROI-based functional activity analyses of the insula. For whole-brain analyses, the minimum cluster size was set at *k* ≥ 100, and for ROI analyses at *k* ≥ 5. Significant results below the cluster threshold are provided in the Supplementary Materials.

## 3 Results

### 3.1 Clinical results

Patients with SAD reported significantly higher scores on all IU subscales and total score (IU-18), as well as on measures of depressive symptoms (BDI-II), state anxiety (STAI-T), and social anxiety symptoms related to situations in which their behavior is or could be observed by others (SPS; *p* ≤ .009). Social anxiety symptoms were positively associated with the IU subscales *prospective IU, inhibitory IU* and *burden due to IU* (*r* ≥ 0.547*, p* < .001) in the SAD group, whereas no significant associations were observed in the HC group (*p* ≥ .06). Hierarchical linear regression analysis in the SAD group indicated that IU significantly predicted social anxiety symptoms (β = 0.513, *p* = .023) and accounted for additional variance beyond age, gender, depressive symptoms, and trait anxiety (ΔR^2^ = 0.145, *p* = .023). In the HC group, adding IU did not significantly increase the explained variance in social anxiety symptoms, and trait anxiety remained the only significant predictor in the final model (β = 0.785, *p* = .004). For detailed results, see Table 3a, Table 3b.

**Table 3a:** Results of the hierarchical linear regression analysis predicting SPS scores in the SAD group (*n* = 25).

Predictor	*B*	*SE B*	β	*t*	*p*	*R*^*2*^	*ΔR*^*2*^	*ΔF*
*Model 1*						0.290	0.290	4.498*
Age	−0.183	0.677	−0.053	−0.270	0.789			
Gender	17.031	6.531	0.514	2.608	0.016			

*Model 2*						0.405	0.115	1.928
Age	0.058	0.666	0.017	0.087	0.931			
Gender	15.242	6.716	0.460	2.270	0.034			
STAI-T	0.809	0.537	0.560	1.506	0.148			
BDI-II	−0.377	0.550	−0.259	−0.685	0.501			

*Model 3*						0.550	0.145	6.139*
Age	−0.495	0.634	−0.144	−0.780	0.445			
Gender	7.006	6.850	0.212	1.023	0.319			
STAI-T	0.276	0.525	0.191	0.526	0.605			
BDI-II	−0.131	0.500	−0.090	−0.261	0.797			
IU-18 Sum	0.477	0.192	0.513	2.478	0.023			

*Note.* SAD, Social Anxiety Disorder; IU-18 Sum, total score for the Uncertainty Intolerance Scale-18 (Gerlach et al., 2008); STAI-T, Spielberger State-Trait Anxiety Inventory (Laux et al., 1981); BDI-II, Beck Depression Inventory II (Beck et al., 1996); SPS, Social Phobia Scale (Ulrich et al., 1999); *p < .05.

**Table 3b:** Results of the hierarchical linear regression analysis predicting SPS scores in the HC group (*n* = 25).

Predictor	*B*	*SE B*	β	*t*	*p*	*R*^*2*^	*ΔR*^*2*^	*ΔF*
*Model 1*						0.053	0.053	0.612
Age	−0.046	0.214	−0.045	−0.213	0.833			
Gender	2.763	2.499	0.235	1.106	0.281			

*Model 2*						0.396	0.343	5.686*
Age	0.030	0.181	0.030	0.165	0.871			
Gender	1.967	2.107	0.167	0.933	0.362			
STAI-T	0.631	0.190	0.795	3.319	0.003			
BDI-II	−0.524	0.286	−0.439	−1.833	0.082			

*Model 3*						0.287	0.039	1.319
Age	−0.019	0.185	−0.019	−0.101	−0.920			
Gender	1.594	2.115	0.135	0.754	0.460			
STAI-T	0.622	0.189	0.785	3.299	0.004			
BDI-II	−0.583	0.288	−0.489	−2.023	0.057			
IU-18 Sum	0.114	0.099	0.217	1.149	0.265			

*Note.* SAD, Social Anxiety Disorder; IU-18 Sum, total score for the Uncertainty Intolerance Scale-18 (Gerlach et al., 2008); STAI-T, Spielberger State-Trait Anxiety Inventory (Laux et al., 1981); BDI-II, Beck Depression Inventory II (Beck et al., 1996); SPS, Social Phobia Scale (Ulrich et al., 1999); *p < .05.

### 3.2 Group differences in functional activity and connectivity

Analyses of group differences in brain activity during face processing revealed no significant findings at the whole-brain level exceeding the minimum cluster size (*k* = 2, *p*_TFCE-FWE_ = 0.048). In the ROI analyses, HC showed higher left insula activity than SAD patients (*k* = 26, T_46_ = 3.81, x = −28, y = 24, z = 24, *p*_TFCE-FWE_ = 0.027, Cohen's *d* = 0.087).

Analyses of group differences in functional connectivity of the left and right insula were not significant (*p*_TFCE-FWE_ > 0.124).

### 3.3 Prospective IU

The analysis of functional activity revealed a significant *prospective IU* x *group* interaction in a cluster comprising the left inferior parietal gyrus (IPG) and supramarginal gyrus (*k* = 206, T_40_ = 4.71, x = −40, y = −50, z = 42, *p*_TFCE-FWE_ = 0.031, Cohen's *f*^2^ = 0.198; see Fig. 1) at the whole-brain level, indicating that the association between *prospective IU* and neural activity during face processing differed between HC and patients with SAD. Follow-up analyses conducted separately for each group revealed a positive association between *prospective IU* and brain activity in the HC group in a cluster comprising the bilateral precuneus, left middle cingulate and paracingulate gyri (*k* = 108, T_40_ = 4.89, x = 4, y = −50, z = 40, *p*_TFCE-FWE_ = 0.033, Cohen's *f*^2^ = 0.264), whereas no significant association was observed in the SAD group (*p*_TFCE-FWE_ = 0.486).

**Fig. 1:**
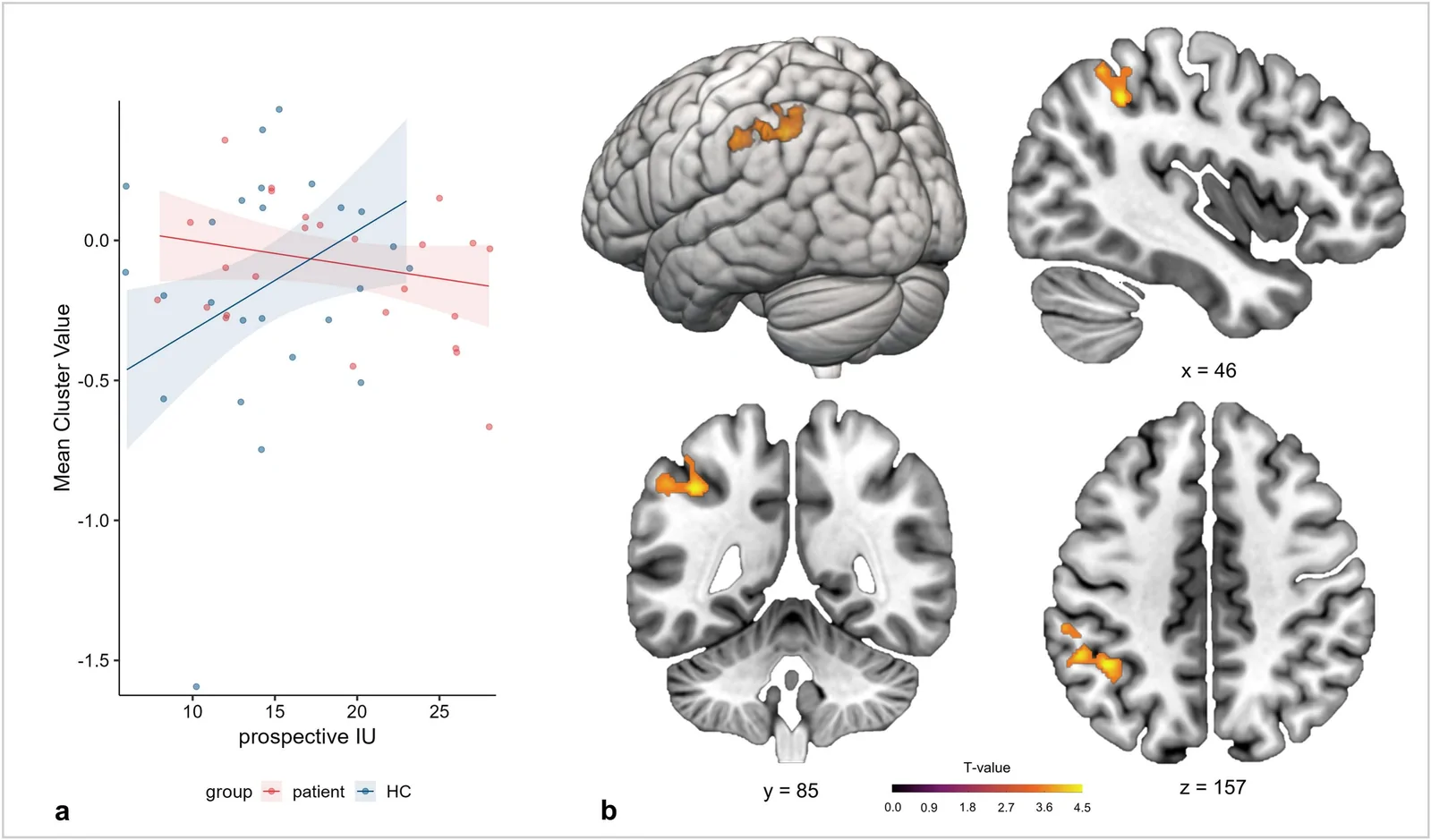
a Scatterplot depicting the correlation of *prospective IU* and functional activity (mean cluster activity value at x = −40, y = −50, z = 42) separated by group; continuous lines represent regression slopes with 95% confidence bands. b Interaction effect of group (SAD, HC) x *prospective IU* in a cluster comprising the left inferior parietal gyrus (IPG) and supramarginal gyrus (*p*_TFCE-FWE_ = 0.031).

ROI analysis also showed a significant interaction (*k* = 126, T_40_ = 4.09, x = −32, y = 20, z = 2, *p*_TFCE-FWE_ = 0.014, Cohen's *f*^2^ = 0.192) in the left insula, with separate group analysis indicating a positive association between *prospective IU* and insula activity only in the HC (*k* = 114, T_40_ = 5.52, x = 46, y = −4, z = 4, *p*_TFCE-FWE_ = 0.003, Cohen's *f*^2^ = 0.122), but not in the SAD group (*p*_TFCE-FWE_ = 0.379).

Seed-based functional connectivity analyses of the left insula demonstrated a significant negative main effect of *prospective IU*, with higher *prospective IU* across groups being associated with decreased functional connectivity between the left insula and seven distinct clusters (0.001 ≤ *p*_TFCE-FWE_ ≤ 0.031, Cohen's *f*^2^ = 0.152). The significant clusters were distributed bilaterally, with a predominance in the right hemisphere, and encompassed primarily superior, middle, and inferior frontal regions, extending into temporal, parietal, striatal, and cingulate areas (see Fig. 2).

**Fig. 2:**
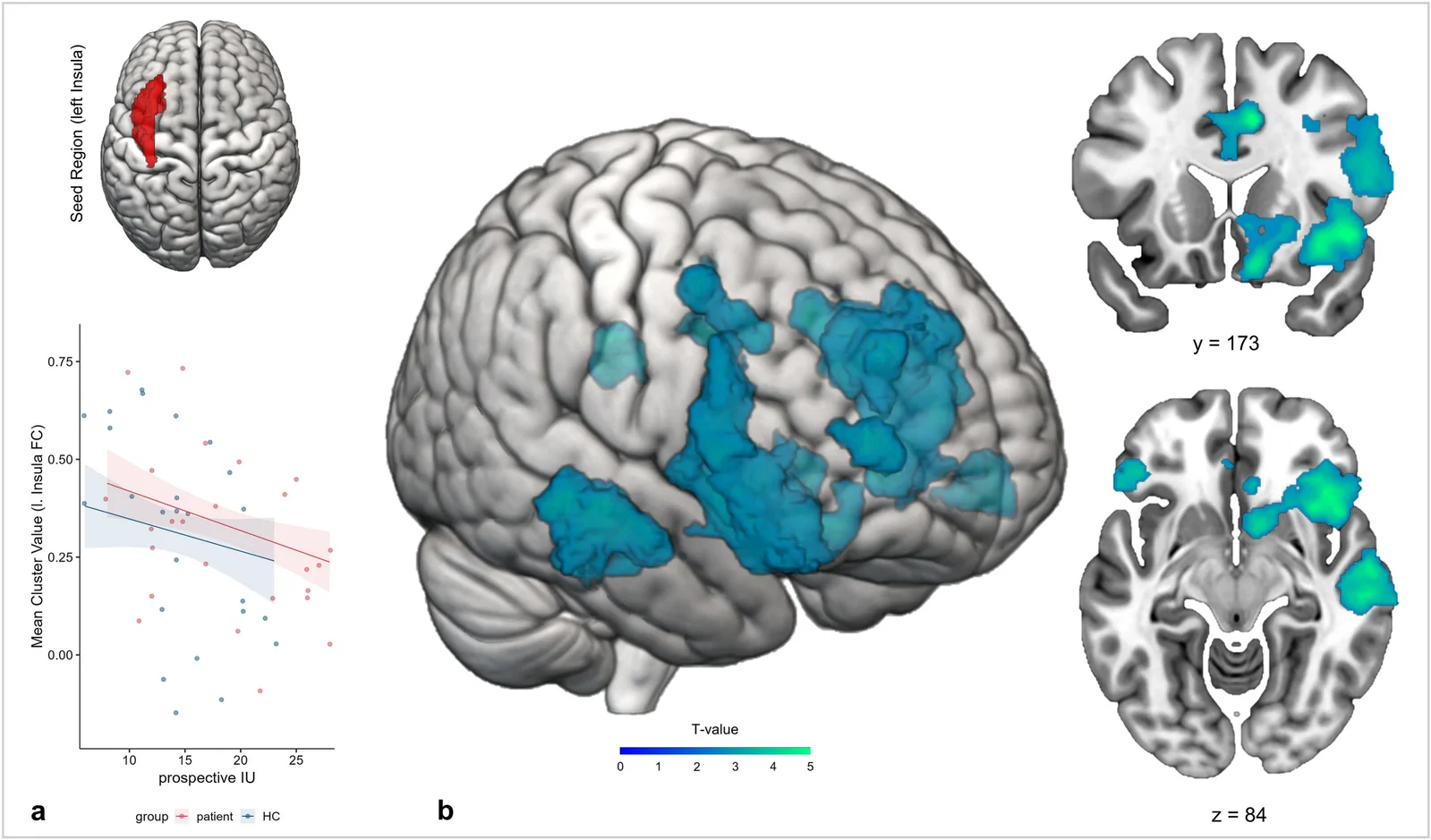
a Scatterplot depicting the correlation of *prospective IU* and insular functional connectivity (mean cluster activity value at x = 46, y = 12, z = 6) separated by group; continuous lines represent regression slopes with 95% confidence bands. b Main effect of *prospective IU* on insular functional connectivity indicating reduced functional coupling between the left insula and seven distinct clusters (0.001 ≤ *p*_TFCE-FWE_ ≤ 0.031).

Functional connectivity analyses using the right insula as the seed region likewise revealed a significant negative main effect of *prospective IU* across eight clusters encompassing anterior and middle cingulate regions, as well as temporal, parietal, and frontal areas (0.010 ≤ *p*_TFCE-FWE_ ≤ 0.031, Cohen's *f*^2^ = 0.103). For detailed results, see Table 4.

**Table 4:** Reduced functional connectivity between left/right insula and significant clusters associated with higher *prospective IU.*

	Side	Cluster Size (*k*)^1^	Peak Voxel Coordinates (MNI)			T-value	*p*_TFCE-FWE_-value
Left Insula (Seed region)							
Superior frontal G., Inferior frontal G., Orbital G., ACC, MCC, Middle frontal G., G. rectus, Insula, Ventral Striatum, Putamen, Olfactory Cortex, Temporal pole of the superior temporal G., Rolandic Operculum, Caudate nucleus, Pallidum	L/R	6265	46	12	6	5.85	0.001
Temporal G.	R	1199	56	−12	−12	4.91	0.005
Middle frontal G.	R	290	28	12	36	4.94	0.015
	R	151	44	56	16	4.40	0.026
Inferior frontal G., Orbital G.	L/R	280	−48	32	−16	4.46	0.022
ACC	L/R	162	12	−42	28	4.75	0.024
MCC	L	116	2	−18	28	4.56	0.031

Right Insula (Seed Region)							
ACC, MCC, Precuneus	L/R	1019	6	−20	30	5.14	0.010
Temporal G., Insula, Heschl's G., Putamen	R	603	38	−8	−2	5.27	0.014
Temporal G.	L	365	−56	−10	−20	5.03	0.016
Superior frontal G., ACC	L	339	−6	40	36	4.65	0.018
Inferior frontal G.	R	311	60	22	8	3.96	0.028
Inferior frontal G., Orbital G.	R	145	−44	34	−8	4.32	0.031
ACC	R	129	10	40	2	4.11	0.030
Rectus, ACC, Olfactory Cortex	R	125	10	24	−18	4.58	0.024
Orbital G., Insula	R	113	30	24	−20	4.90	0.020

*Note.* MNI, Montreal Neurological Institute; TFCE, Threshold Free Cluster Enhancement; L, left; R, right; ^1^Clusters with a minimum cluster size *k* ≥ 100 and brain regions that were represented in the cluster with ≥2% of their total size are reported; G., gyrus; SFG, superior frontal gyrus; IFG, inferior frontal gyrus; MFG, Middle Frontal Gyrus; ACC, Anterior Cingulate Cortex; MCC, Middle Cingulate Gyrus.

### 3.4 Burden due to IU

Functional activity analyses at the whole-brain level revealed a significant negative main effect of *burden due to IU* on brain activity in a cluster encompassing the bilateral precuneus and posterior cingulate gyrus (PCC; *k* = 153, T_40_ = 5.44, x = −4, y = −46, z = 22, *p*_TFCE-FWE_ = 0.014, Cohen's *f*^2^ = 0.017); see Fig. 3), indicating a negative association between *burden due to IU* and functional activity across groups.

**Fig. 3:**
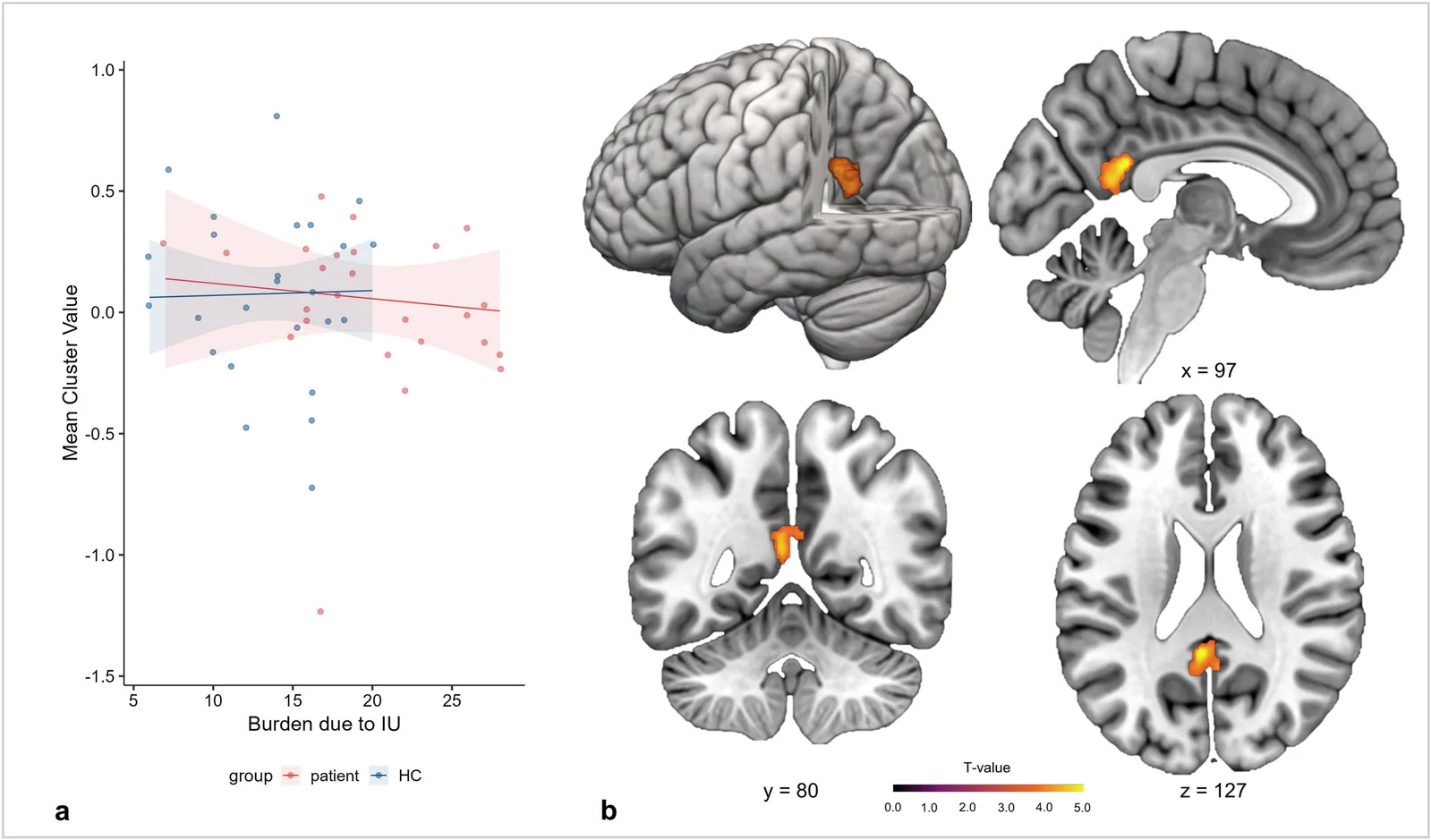
a Scatterplot depicting the correlation of *burden due to IU* and functional activity (mean cluster activity value at x = −4, y = −46, z = 22) separated by group; continuous lines represent regression slopes with 95% confidence bands. b Main effect of *burden due to IU* on brain activity in a cluster comprising the bilateral precuneus and posterior cingulate gyrus (*p*_TFCE-FWE_ = 0.014).

ROI analysis also revealed a significant negative main effect on the right insula (*k* = 49, T_40_ = 4.42, x = 46, y = −2, z = 2, *p*_TFCE-FWE_ = 0.013, Cohen's *f*^2^ = 0.044), indicating a negative association between *burden due to IU* and insula activity across groups.

Functional connectivity analyses did not reveal any significant effects of *burden due to IU* on left or right insula connectivity exceeding the minimum cluster size threshold (*k* = 4, *p*_TFCE-FWE_ = 0.041).

### 3.5 Inhibitory IU

Functional activity analyses did not reveal any significant effects of *inhibitory IU* on whole-brain or insula activity exceeding the minimum cluster size threshold (*k* = 4, *p*_TFCE-FWE_ = 0.044).

Likewise, functional connectivity analyses did not reveal any significant effects of *inhibitory IU* on left or right insula connectivity exceeding the minimum cluster size threshold (*k* = 67, *p*_TFCE-FWE_ = 0.024).

### 3.6 IU sum scale

The described analyses were repeated using the total score of the IU-18 questionnaire. Functional activity analyses on whole-brain or insula activity did not reveal any significant effects (*p*_TFCE-FWE_ > 0.072), nor did analyses of functional connectivity (*p*_TFCE-FWE_ > 0.102).

### 3.7 Associations of social anxiety symptoms with functional activity and connectivity

Whole-brain analysis of functional activity revealed a significant *SPS* x *group* interaction in seven clusters (0.005 ≤ *p*_TFCE-FWE_ ≤ 0.027; Cohen's *f*^2^ = 0.364). Significant clusters were distributed bilaterally across superior, middle, and inferior frontal regions, extending into temporal, parietal, striatal, and cingulate regions, as well as the cerebellum.

Follow-up analyses conducted separately for each group revealed a positive association between SPS and brain activity in the HC group in seven clusters (0.008 ≤ *p*_TFCE-FWE_ ≤ 0.041, Cohen's *f*^2^ = 0.482), whereas no significant association was observed in the SAD group (*p*_TFCE-FWE_ = 0.782), indicating that the interaction effect was mainly driven by the positive association in the HC group.

ROI analysis of the bilateral insula did not reveal any significant associations (*p* ≥ .057).

Functional connectivity analyses using the left and right insula as seed regions also yielded no significant associations between SPS scores and functional connectivity of either insular seed (*p* ≥ .117). For detailed results, see Supplementary Table 1.

## 4 Discussion

Extending previous work on SAD, emotional face processing, and IU, the present study sought to elucidate dimensional associations between IU subdimensions, neural activity, and insular functional connectivity during repeated exposure to angry and fearful faces. Central findings of our analyses were distinct group-specific as well as cross-group association patterns for the IU subdimensions *prospective IU,* and *burden due to IU* with brain activity and insular functional connectivity.

Concerning *prospective IU*, the cognitive and future-oriented subdimension of IU, a significant negative main effect was found for bilateral insular functional connectivity, involving extensive clusters across frontal, cingulate, temporal, and subcortical regions. This suggests that across both the SAD and HC groups, elevated levels of *prospective IU* were associated with reduced functional coupling of the insula with distributed cortical and subcortical regions involved in executive control, salience, and affective-regulatory networks (Menon and D'Esposito, 2022). As a hub region embedded in multiple large-scale networks, particularly the salience and default mode, the insula plays a key mediating role in guiding attentional and interoceptive processes (Menon and Uddin, 2010). The observed reduction in insular functional connectivity with regions spanning these and other networks may therefore reflect an IU-related attenuation of this integrative function, consistent with findings reported by Deserisy et al. (2020), who identified prospective IU-related reductions in insular network coupling and Pantazatos et al. (2014), who reported reduced functional connectivity during face perception in individuals with SAD. A reduction of insular integration may compromise affective processing in response to a potential threat induced by negative emotional stimuli, operating independently of SAD diagnosis.

Concurrently, analyses of brain activity revealed an interaction between *prospective IU* and group, indicating that the association between this IU subdimension and neural responses to emotional faces differed as a function of SAD. The interaction effect emerged at the whole-brain level in left-lateralized parietal regions, specifically within the inferior parietal (IPG) and supramarginal gyrus. These regions are canonically implicated in somatosensory and semantic processing (Numssen et al., 2021; Oberhuber et al., 2016), but have more recently been associated with functions such as empathy (Kogler et al., 2020) and emotional contagion (Fu et al., 2025). At the ROI level, a significant interaction effect was additionally observed in the left insula, which was previously found to be co-activated with the IPG during emotional resonance (Fu et al., 2025). Post-hoc subgroup analyses indicated that these effects were each driven by a positive association between *prospective IU* and brain activity in the HC group, while no significant association was detected in the SAD group.

Integrating the findings on insula connectivity and functional activity, the results may suggest that *prospective IU* is associated with reduced functional insula integration independent of diagnostic status, while being selectively linked to increased neural activity in the HC group. The heightened activation in regions implicated in social perspective-taking and empathy (IPG, supramarginal gyrus), as well as uncertainty processing (insula), may reflect an adaptive, trait-modulated upregulation of neural responses in healthy individuals. A potential interpretation is that, as the need to reduce uncertainty through cognitive processes (i.e., *prospective IU*) increases, there is a corresponding recruitment of brain regions implicated in empathetic reasoning, which may support the resolution of social threat conveyed by negative emotional stimuli. These findings may reflect a compensatory mechanism for diminished insular functional connectivity in healthy adults.

An IU-related increase in brain activity was not observed in the patient group, which may at first glance not directly align with well-established findings of heightened neural responses to emotional facial expressions (Carré et al., 2014; Gentili et al., 2016; Klumpp et al., 2011) and other disorder-relevant stimuli (Heitmann et al., 2016) in individuals with SAD, typically interpreted within a framework of hyperactive fear circuitry and enhanced salience attribution to socially threatening cues. The insula, in particular, has frequently been reported to exhibit hyperactivity in SAD (Marazziti et al., 2015), a finding that may reflect heightened self-focused attention and interoception, in line with the core symptomatology of the disorder. However, this association was not observed in the present study. In addition, SAD patients showed lower left insula activity than HC at the group level. Although this result differs from previous findings of heightened insula activity in SAD, it may point to a context-specific alteration in insula functioning, as well as to a lack of additional modulation of neural responses by trait-level constructs such as *prospective IU*, which was observed in the HC group. Accordingly, in SAD, there may be limited capacity to recruit compensatory mechanisms to counteract reduced insula integration. Given the region's central role in processing and coping with uncertainty, this may contribute to disorder-specific impairments in adapting to the inherent uncertainty of social stimuli, potentially promoting the avoidance behavior characteristic of SAD.

Analyses of the subdimension *burden due to IU*, reflecting affective distress under uncertainty, revealed another distinct association pattern. There were no significant effects regarding functional connectivity; however, across groups, *burden due to IU* was negatively associated with whole-brain activity in the bilateral precuneus and PCC, as well as with right insular activity at the ROI-level. Similar to the insula, the precuneus and the PCC are regarded as highly interconnected regions, and contribute to higher-order cognitive processes by integrating mnemonic, sensory and self-referential information (Cavanna and Trimble, 2006; Dadario and Sughrue, 2023; Foster and Koslov, 2025). With higher IU-related distress, activity in these integrative brain regions decreases during the processing of negative emotional faces, which may indicate a relatively reduced capacity to contextualize uncertainty inherent in social-emotional stimuli.

Furthermore, given that the precuneus and PCC are key nodes within DMN subnetworks implicated in mentalizing and theory of mind processes (Hyatt et al., 2015), and are consistently co-activated during social cognition tasks (Schilbach et al., 2012), their relative deactivation in the present task may implicate a broader IU-related difficulty in inferring others' mental states and engaging in perspective taking. Tentatively, it may be inferred that greater *burden due to IU* is associated with a diminished capacity to engage in mentalizing when confronted with negative emotions, across both clinical and non-clinical groups.

*Inhibitory IU* was not significantly associated with brain activity or insular functional connectivity in either group. Since this IU subdimension captures behavioral inhibition in response to uncertainty, it may represent a secondary outcome of uncertainty processing rather than an immediate cognitive or affective response (Gerlach et al., 2008). Moreover, as the paradigm employed in the present study was not specifically designed to elicit or assess behavioral inhibition, this may further explain the absence of associations with *inhibitory IU* across groups.

Lastly, additional analyses using the Social Phobia Scale (SPS) score as the independent variable in the full-factorial model revealed a similar pattern of associations with whole-brain activity as for *prospective IU*. A significant interaction effect, driven by a positive association between SPS scores and brain activity in the HC group, was observed across widespread clusters encompassing frontal, temporal, parietal, striatal, and cingulate regions, with extensions into the cerebellum. Given that the SPS specifically assesses discomfort in situations involving observation and potential evaluation by others, higher SPS scores may be associated with increased neural responsiveness to the facial stimuli used in the present paradigm. As this association again differed as a function of SAD diagnosis, with a positive association significant in the HC group, this pattern may reflect an adaptive modulation of neural responses in individuals without SAD or a reduced differentiation of neural responses as a function of SPS scores in individuals with SAD.

### 4.1 Implications

Clinical implications may be particularly derived from the group-differential pattern observed for *prospective IU*, which revealed both shared and disorder-specific neural associations. The finding that healthy individuals exhibited a potentially adaptive association between *prospective IU* and neural regions implicated in social cognition, which was not observed in the SAD group, suggests that targeting the perception and interpretation of social cues may be an important component in the treatment of SAD. This reasoning aligns with emerging evidence suggesting that therapeutic approaches addressing relational functioning in social anxiety may yield beneficial treatment outcomes (Alden et al., 2018). Given the differential neural profiles associated with the IU subdimensions, it may be clinically valuable to assess not only the overall level of IU a patient experiences but also how they individually process, react to, and attempt to resolve uncertainty, particularly in social situations. These individual profiles may identify maintaining processes in SAD psychopathology and, accordingly, distinct points for therapeutic leverage. Findings from the hierarchical regression analyses, showing that IU significantly predicted social anxiety symptoms and explained additional variance only in the SAD group, further underscore the relevance of this construct in the context of SAD.

From a theoretical perspective, the differential association patterns observed across IU subscales, alongside the absence of associations for the IU sum scale, may support the conceptualization of IU as a multifaceted construct rather than a unitary factor, as advocated for by previous research (Bottesi et al., 2019; Hale et al., 2016). Although a two-factor conceptualization currently represents the most widespread operationalization of IU, our findings on the subdimension *burden due to IU* highlight the potential relevance of further addressing this distinct facet in future research. In particular, this dimension may capture the emotional strain associated with experiencing uncertainty and thereby provide complementary information beyond the cognitive and behavioral components reflected in the *prospective* and *inhibitory IU* dimensions.

### 4.2 Strengths and limitations

To our knowledge, this is the first study to investigate the influence of IU on brain activity and functional connectivity during emotional face processing in SAD, employing a well-established experimental paradigm. By examining the distinct associations of IU subdimensions, including the relatively understudied *burden due to IU* component, this approach enables a more nuanced understanding of how IU may interact with disorder-specific processes. Integrating measures of regional activation and functional connectivity may provide a mechanistic perspective on how localized neural responses and broader network-level integration jointly contribute to the pathophysiology of SAD.

However, the modest sample size and cross-sectional design, which preclude causal inferences about the association between IU and brain activity and connectivity, should be considered when interpreting the statistical power and significance of the findings. Larger sample sizes are required to replicate the present results. Although *burden due to IU* constitutes a theoretically well-founded subdimension of IU, its investigation and psychometric assessment are currently limited to the German translation of the IU scale originally introduced by Freeston et al. (1994), and as such, has not yet undergone extensive validation.

### 4.3 Future lines of research

Future lines of research should consider additional seed regions beyond the insula, as prior studies of network connectivity in SAD have implicated the involvement of other structures, such as the hippocampus (Pantazatos et al., 2014). Furthermore, given the evidence for distinct neural correlates for angry versus fearful faces (Klumpp et al., 2011) in SAD, future studies may benefit from differentiating between emotional expressions when selecting experimental stimuli. In particular, the processing of ambiguous emotional stimuli may be especially sensitive to individual differences in IU and therefore warrants further investigation. Finally, longitudinal studies should examine whether changes in IU levels among individuals with SAD over the course of psychotherapy are accompanied by corresponding changes in neural activity and functional connectivity.

## 5 Conclusion

The present study aimed to elucidate the interplay between IU subdimensions and neural activity during emotional face processing in individuals with and without a SAD diagnosis, revealing an intricate pattern of both cross-group and group-specific associations.

The distinct neural association patterns observed across the *prospective IU* and *burden due to IU* subdimensions support the conceptualization of IU as a multifaceted construct (Gerlach et al., 2008) whose components carry differential implications in the context of psychopathology. These findings underscore the value of incorporating IU not only as a general factor, but with respect to its subdimensions in neuroimaging research, potentially allowing for a more nuanced characterization of the cognitive and affective processes contributing to dysfunctional stimulus processing in SAD.

## CRediT authorship contribution statement

**Antonia Küttner:** Writing – original draft, Project administration, Methodology, Investigation, Formal analysis, Data curation, Conceptualization. **Esther Zwiky:** Writing – review & editing, Project administration, Methodology, Investigation, Data curation. **Philine König:** Writing – review & editing, Project administration, Investigation, Conceptualization. **Konrad Schöniger:** Writing – review & editing, Methodology, Investigation, Data curation. **Robin Kamrla:** Writing – review & editing, Software, Methodology, Investigation, Data curation. **Gina Sophie Scheiwe:** Writing – review & editing, Investigation. **Julia Andreev:** Writing – review & editing, Investigation. **Johanna Elea Hamann:** Writing – review & editing, Investigation. **Annika Brückner:** Writing – review & editing, Investigation. **Sina Pletsch:** Writing – review & editing, Investigation. **Janine Selle:** Writing – review & editing, Investigation. **Alexander Gussew:** Writing – review & editing, Software, Resources, Methodology. **Andreas Deistung:** Writing – review & editing, Software, Resources, Methodology. **Walter Alexander Wohlgemuth:** Writing – review & editing, Project administration, Funding acquisition, Conceptualization. **Ronny Redlich:** Writing – review & editing, Supervision, Project administration, Funding acquisition, Conceptualization.

## Declaration of competing interest

The authors declare that they have no known competing financial interests or personal relationships that could have appeared to influence the work reported in this paper.

## Data Availability

Data will be made available on request.
