# Supplementary material for: Why are you looking at me like that? Intolerance of uncertainty and emotional face processing in social anxiety disorder
Source: Neuroimage Clin. 2026 Sep 25;52:104069. doi: 10.1016/j.nicl.2026.104069 (PMC13651650; doi:10.1016/j.nicl.2026.104069)
Supplement: Supplementary file 1 [file mmc1.docx]

**Supplementary Materials**

*Why are you looking at me like that? Intolerance of Uncertainty and Emotional Face Processing in Social Anxiety Disorder*

# 1. Supplementary Methods

## 1.1 Preprocessing for functional connectivity analyses

To investigate alterations in insular functional connectivity during emotional face processing, fMRI data were additionally preprocessed using the CONN toolbox^1^ implemented within a modular preprocessing pipeline.

Preprocessing of fMRI data included realignment with correction of susceptibility distortion interactions, slice timing correction, outlier detection, direct segmentation and MNI-space normalization, and smoothing. Functional data were realigned using SPM realign & unwarp procedure, where all scans were coregistered to a reference image (first scan of the first session) using a least squares approach and a 6 parameter (rigid body) transformation, and resampled using b-spline interpolation to correct for motion and magnetic susceptibility interactions. Temporal misalignment between different slices of the functional data was corrected following SPM slice-timing correction (STC) procedure, using sinc temporal interpolation to resample each slice BOLD timeseries to a common mid-acquisition time. Potential outlier scans were identified using ART^2^ as acquisitions with framewise displacement above 0.9 mm or global BOLD signal changes above 5 standard deviations, and a reference BOLD image was computed for each subject by averaging all scans excluding outliers. Functional and anatomical MRI data were normalized into standard MNI space, segmented into grey matter, white matter, and CSF tissue classes, and resampled to 2 mm isotropic voxels following a direct normalization procedure using SPM unified segmentation and normalization algorithm with the default IXI-549 tissue probability map template. Last, functional data were smoothed using spatial convolution with a Gaussian kernel of 8 mm full width half maximum (FWHM). In addition, functional data were denoised using a standard denoising pipeline including the regression of potential confounding effects characterized by white matter timeseries (5 CompCor^3^ noise components), CSF timeseries (5 CompCor noise components), motion parameters and their first order derivatives (12 factors), outlier scans (below 8 factors), session and task effects and their first order derivatives (4 factors), and linear trends (2 factors) within each functional run, followed by bandpass frequency filtering of the BOLD timeseries between 0.01 Hz and 0.1 Hz. CompCor noise components within white matter and CSF were estimated by computing the average BOLD signal as well as the largest principal components orthogonal to the BOLD average, motion parameters, and outlier scans within each subject's eroded segmentation masks. From the number of noise terms included in this denoising strategy, the effective degrees of freedom of the BOLD signal after denoising were estimated to range from 56.4 to 58.8 (average 58.2) across all subjects. For the first-level analysis, seed-based connectivity maps (SBC) and ROI-to-ROI connectivity matrices (RRC) were estimated from preprocessed and denoised fMRI data characterizing the patterns of functional connectivity with 164 HPC-ICA networks and Harvard-Oxford atlas ROIs. Functional connectivity strength was represented by Fisher-transformed bivariate correlation coefficients from a weighted general linear model (weighted-GLM), defined separately for each pair of seed and target areas, modeling the association between their BOLD signal timeseries. Individual scans were weighted by a boxcar signal characterizing each individual task or experimental condition, convolved with an SPM canonical hemodynamic response function, and rectified.

# 2. Supplementary Results

## 2.1 Subthreshold Functional Activity Results

### 2.1.1 Group differences in functional activity

Analyses of group differences in brain activity during face processing at the whole-brain level revealed higher brain activity in HC compared to SAD patients in the left supplementary motor area (*k* = 2, T_46_ = 4.71, x = -6, y = 4, z = 56, *p*_TFCE-FWE_ = .048).

## 2.2 Subthreshold Functional Connectivity Results

### 2.2.1 Burden due to IU

Functional connectivity analyses revealed a positive main effect of *burden due to IU* on functional coupling between the left insula and the right middle cingulate gyrus (*k* = 4, T_40_ = 6.04, x = 8, y = 16, z = 36, *p*_TFCE-FWE_ = .041).

### 2.2.2 Inhibitory IU

Functional connectivity analyses revealed an *inhibitory IU x group* interaction in three clusters, comprising the left superior and middle frontal gyrus (*k* =67, T_40_ = 5.13, x = -26, y = 60, z = 28, *p*_TFCE-FWE_ = .024), the bilateral medial superior frontal gyrus (*k* = 34, T_40_ = 4.58, x = 0, y = 28, z = 52, *p*_TFCE-FWE_ = .037) and the right supracallosal anterior and middle cingulate cortex (*k* = 25, T_40_ = 4.72, x = 14, y = 32, z = 24, *p*_TFCE-FWE_ = .039). Follow-up analyses conducted separately for each group revealed a positive association between *inhibitory IU* and brain activity in the HC group (*k* = 145, T_40_ = 6.23, x = -24, y = 60, z = 28, *p*_TFCE-FWE_ = .016; cluster comprising the left precuneus and left superior and middle frontal gyrus), whereas no significant association was observed in the SAD group (*p*_TFCE-FWE_ = .790).

## 2.3 Associations of social anxiety symptoms with functional activity and connectivity

Detailed results for the *SPS x group* interaction effect as well as the subgroup analyses are presented below in Supplementary Table 1.

**Supplementary Table 1.**

|  | Side | Cluster Size (*k*)^1^ | Peak Voxel Coordinates (MNI) | | | T-value | *p*_TFCE-FWE_-value |
| --- | --- | --- | --- | --- | --- | --- | --- |
| ***SPS x group* interaction on whole-brain functional activity** | | | | | | | |
| MFG, IFG, SFG, Supplementary Motor Area, Precentral G., MCC, Temporal Pole, Superior Temporal G., Paracentral Lobule, Insula, Precuneus, ACC, Heschl’s G., Rolandic Operculum, Putamen, Lateral Orbital G., Pallidum | L/R | 10145 | 48 | 14 | 30 | 4.35 | .005 |
| Parietal G., Angular G. | L | 799 | -36 | -72 | 50 | 3.68 | .025 |
| Crus II and Lobule VI of Cerebellar Hemisphere, Lobule VII of Vermis | L/R | 450 | -4 | -80 | -30 | 4.12 | .020 |
| Putamen, Caudate nucleus, Temporal Pole, Posterior Orbital G., Pallidum | L | 416 | -16 | 12 | 4 | 3.59 | .025 |
| Temporal G. | L | 375 | 60 | -50 | -4 | 3.78 | .026 |
| Cuneus, Calcarine fissure | L | 151 | 2 | -90 | 20 | 5.11 | .016 |
| Inferior Parietal G. | R | 119 | 32 | -50 | 44 | 3.94 | .027 |
| **Association between SPS and whole-brain activity in HC group** | | | | | | | |
| SFG, MCC, Supplementary Motor Area, MFG, Precentral G., Precuneus, Paracentral Lobule | L/R | 5347 | -4 | -40 | 56 | 5.05 | .008 |
| MFG, Precentral G. | L | 544 | -42 | 10 | 52 | 4.08 | .028 |
| IFG | L | 323 | -40 | 30 | 4 | 4.11 | .035 |
| MFG, IFG | R | 316 | 46 | 44 | 16 | 4.59 | .024 |
| Calcarine Fissure, Cuneus | L | 208 | 2 | -90 | 20 | 5.27 | .018 |
| IFG, Lateral Orbital G. | R | 137 | 46 | 28 | -10 | 3.88 | .041 |
| Supplementary Motor Area | R | 119 | 4 | -8 | 70 | 4.20 | .031 |

Association between SPS and whole-brain activity: results from the analysis of *SPS x group* interaction on functional activity and subgroup analysis in the HC group

*Note.* MNI, Montreal Neurological Institute; TFCE, Threshold Free Cluster Enhancement; L, left; R, right; HC, healthy controls; ^1^Clusters with a minimum cluster size *k* ≥ 100 and brain regions that were represented in the cluster with ≥ 2% of their total size are reported; G., gyrus; SFG, superior frontal gyrus; IFG, inferior frontal gyrus; MFG, Middle Frontal Gyrus; ACC, Anterior Cingulate Cortex; MCC, Middle Cingulate Gyrus.

# 3. References

1.	Whitfield-Gabrieli, S. & Nieto-Castanon, A. Conn: A Functional Connectivity Toolbox for Correlated and Anticorrelated Brain Networks. *https://home.liebertpub.com/brain* **2**, 125–141 (2012).

2.	Whitfield-Gabrieli, S., Nieto-Castanon, A. & Ghosh, S. Artifact detection tools (ART). Preprint at (2011).

3.	Behzadi, Y., Restom, K., Liau, J. & Liu, T. T. A component based noise correction method (CompCor) for BOLD and perfusion based fMRI. *Neuroimage* **37**, 90–101 (2007).
